# Activation patterns of rotator-cuff muscles from quantitative IVIM DWI after physical testing

**DOI:** 10.1186/s41747-024-00487-5

**Published:** 2024-08-26

**Authors:** Adrian Alexander Marth, Georg Ralph Spinner, Constantin von Deuster, Stefan Sommer, Reto Sutter, Daniel Nanz

**Affiliations:** 1 Swiss Center for Musculoskeletal Imaging (SCMI), Balgrist Campus AG, Zurich, Switzerland; 2 Department of Radiology, Balgrist University Hospital, Zurich, Switzerland; 3 Center of Computational Health, Institute of Computational Life Sciences, University of Applied Sciences (ZHAW), Wädenswil, Switzerland; 4 Advanced Clinical Imaging Technology, Siemens Healthineers International AG, Zurich, Switzerland; 5 Medical Faculty, University of Zurich (UZH), Zurich, Switzerland

**Keywords:** Healthy volunteers, Magnetic resonance imaging, Muscles, Rotator cuff, Shoulder

## Abstract

**Background:**

The diagnostic value of clinical rotator cuff (RC) tests is controversial, with only sparse evidence available about their anatomical specificity. We prospectively assessed regional RC muscle activation patterns by means of intravoxel incoherent motion (IVIM) diffusion-weighted magnetic resonance imaging (MRI) after the execution of common clinical RC tests.

**Methods:**

Ten healthy subjects (five males, five females) underwent three sessions of diffusion-weighted 3-T shoulder MRI before and after testing the supraspinatus (SSP, Jobe test, session 1), subscapularis (SSC, lift-off test, session 2, at least 1 week later), and infraspinatus muscle (ISP, external rotation test, session 3, another week later). IVIM parameters (perfusion fraction, *f*; pseudo-diffusion coefficient. D*; and their product, fD*) were measured in regions of interest placed in images of the SSP, SSC, ISP, and deltoid muscle. The Wilcoxon signed-rank test was used for group comparisons; *p*-values were adjusted using the Bonferroni correction.

**Results:**

After all tests, fD* was significantly increased in the respective target muscles (SSP, SSC, or ISP; *p* ≤ 0.001). After SSP testing, an additional significant increase of fD* was observed in the deltoid, the SSC, and the ISP muscle (*p* < 0.001). After the SSC and ISP tests, no significant concomitant increase of any parameter was observed in the other RC muscles.

**Conclusion:**

IVIM revealed varying activation patterns of RC muscles for different clinical RC tests. For SSP testing, coactivation of the deltoid and other RC muscles was observed, implying limited anatomical specificity, while the tests for the SSC and ISP specifically activated their respective target muscle.

**Relevance statement:**

Following clinical RC tests, IVIM MRI revealed that SSP testing led to shoulder muscle coactivation, while the SSC and ISP tests specifically activated the target muscles.

**Key Points:**

- In this study, intravoxel incoherent motion MRI depicted muscle activation following clinical rotator cuff tests.
- After supraspinatus testing, coactivation of surrounding shoulder girdle muscles was observed.
- Subscapularis and infraspinatus tests exhibited isolated activation of their respective target muscles.

**Graphical Abstract:**

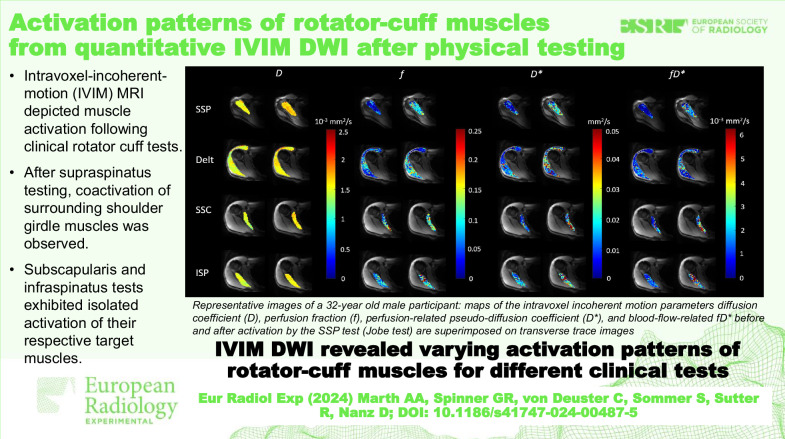

## Background

Rotator cuff (RC) tears are a common health burden in the elderly population and can result in pain and limited function [1, 2]. To detect RC tears, an initial clinical examination is usually done to test for the weakness of the RC muscles [3]. While a variety of corresponding tests exist, the most commonly used tests include the Jobe test for the supraspinatus muscle (SSP) [4, 5], the lift-off test for the subscapularis muscle (SSC), and the external rotation test for the infraspinatus muscle (ISP) [6, 7]. Because of the anatomic complexity of the shoulder, each muscle serves multiple functions which frequently overlap [8]. Thus, some clinical RC tests are likely to not only activate the target muscle but also the surrounding muscles [9, 10], which may result in limited diagnostic accuracy for RC tears, and might potentially influence the assessment of clinical outcomes after RC repair [11].

Electromyography (EMG) studies have demonstrated that different examination positions can influence the extent of muscle coactivation in the RC other than the targeted muscle [9, 12–15]. However, EMG reproducibility is considered to be limited, mostly due to the fact that it detects the activity of a small number of muscle fibers only [16]. On the other hand, quantitative magnetic resonance imaging (MRI) offers noninvasive methods for characterizing exercise-induced regional microstructural changes in skeletal muscle [17]. Among those is intravoxel incoherent motion (IVIM) diffusion-weighted imaging (DWI) MRI, which describes the signal decay for increasing diffusion-sensitization (*b*-value) of the DWI sequence with a biexponential, *i.e.*, two-compartment model, with three parameters: a diffusion coefficient *D* that is assumed to characterize the restricted and averaged thermal self-diffusion of extravascular tissue water; a pseudo-diffusion coefficient D*, assumed to reflect an averaged blood-flow in the network of capillary vessels (microcirculation) that are presumed to have an isotropic orientation distribution; and the perfusion fraction *f*, which characterizes the signal fraction contributed by the flowing capillary blood. Furthermore, the product fD* is thought to correlate with total microvascular blood-flow (perfusion) in the capillary network [18, 19]. Even though it has been demonstrated that IVIM can depict perfusion differences in the skeletal muscle after activation [20], there is little knowledge about activation patterns after physical testing of the SSP and SSC muscle [21, 22], while no activation patterns have yet been described after physical testing of the ISP muscle.

Therefore, the aim of this study was to describe the regional RC muscle activation after performing common clinical tests of the SSP, SSC, and ISP muscle using IVIM imaging and to evaluate possible coactivation of muscles in the surrounding shoulder girdle beyond the targeted muscle.

## Methods

### Participants

The local institutional review board (Cantonal Ethics Commission Zurich, Switzerland) granted approval for this prospective study. Ten healthy volunteers, without a history of any known musculoskeletal pathology (including spine pathology, muscle injury, muscle disease) or prior shoulder surgery, were enrolled after providing written informed consent. Exclusion criteria included the presence of fever at the examination timepoint and incidental detection of any shoulder pathology during the baseline MRI. The research adhered to ethical standards set by the institutional and/or national ethics committee. Participants underwent a total of three examinations, each including a baseline MRI, a clinical shoulder test, and a post-activation MRI. The examinations took place between May and August 2023, with an interval of 1 week between each examination. Participants were acclimatized to the temperature of the MRI examination room, which was 21 °C. Female participants were examined only in the post-ovulatory phase to minimize the effect of sex-related inherent body temperature differences.

### MRI protocol and clinical shoulder examination

MRI data were acquired on a 3-T system (MAGNETOM Prisma, Siemens Healthineers, Erlangen, Germany) using a dedicated 16-channel shoulder coil in the supine position. First, a transverse T1-weighted turbo spin echo sequence was acquired for anatomical reference. The subsequent IVIM acquisition employed a transverse fat-signal suppressing monopolar-pulsed single-shot echoplanar diffusion-weighted sequence that acquired data at multiple *b*-values (0, 20, 40, 60, 80, 100, 200, 300, 400, 500, 600, 700, 800 s/mm^2^) in three orthogonal diffusion-encoding directions, from which the trace was computed. The sequence parameters of the protocol are specified in Table 1.

**Table 1:** Sequence acquisition parameters

Acquisition parameters	T1-weighted	Diffusion-weighted
Repetition time (ms)	700	6,700
Echo time (ms)	11	42
Slice thickness (mm)	3.0	4.0
Number of slices	23	35
Reconstructed matrix size	576 × 576	64 × 64
Field of view (mm^2^)	192 × 192	192 × 192
Parallel imaging acceleration factor	2	2
Receive bandwidth (Hz pixel^−1^)	230	1.302
Acquisition time (min:s)	03:12	08:35

Both sequences were acquired in transverse orientation

After the acquisition of MRI sequences at rest, participants underwent muscle activation by clinical shoulder examination in the MRI scanner room. For the SSP, the arm was abducted to 90°, then angled forward 30°, and internally rotated with the thumb pointing to the floor. The participant then resisted the examiner’s downward pressure on his/her arm (Jobe test) [4]. For the SSC, the participant was instructed to move the hand around the back to the lumbar region with the palm facing outward and attempted to lift his hand off his back against the examiner’s resistance (lift-off test) [7]. For the ISP, the participant’s elbow was flexed 90° and then internally rotated 45° with a towel roll placed under the participant’s armpit. The participant was then instructed to externally rotate the arm against resisted pressure from the examiner while holding the towel in place (external rotation test) [23]. The examinations were conducted with the application of maximum tolerable resisted pressure for two minutes while keeping the participant’s arm in position, as previously described by Nguyen et al [22]. After the clinical shoulder examination, the participants immediately underwent a second MRI scan.

### IVIM image processing

The biexponential signal equation model was assumed for the normalized decay of the diffusion-weighted signal as a function of *b*-value:

1 $$\frac{S\,(b)}{{S}_{0}}={{{{\rm{f e}}}}}^{-b{D}^{* }}+(1-{{{{\rm{f}}}}}){{{{\rm{e}}}}}^{-{bD}}$$

in which *S*_0_ is equilibrium magnetization at *b* = 0 s/mm^2^ [24]. Maps of IVIM parameters were created voxel-wise in the regions of interest (ROIs) and additionally, IVIM parameters were estimated from magnitude averages over all voxels in the respective ROIs by fitting the signal to Equation [1] in two steps using a custom-written Matlab script (version R2023a, Mathworks, Natick, MA) that invoked Matlab’s built-in Trust-Region-Reflective non-linear least-square fitting algorithm (“lsqnonlin”). In the first step, a mono-exponential function was fitted to the signal-decay curve for *b*-values of 200 s/mm^2^ and larger, to obtain parametric maps of *D* and of the *y*-intercept *S*_*0,high*_. In a second step, the Equation [1] was fitted to the signal-decay curve, including data acquired at all *b*-values, searching for the best values for *f*, D*, and *S*_0_ while keeping D and *S*_*0,high*_ = *S*_0_ (1 − f ) at constant values obtained from the first step.

### Quantitative image analysis

ROIs were manually drawn over the *b* = 0 s/mm^2^ images on the slice in which the maximum cross-sectional area of the respective muscle of interest was visible (SSP, deltoid, SSC, ISP), attempting to exclude tendons and fasciae. In order to assess the activation of the tested muscles, absolute intramuscular changes in IVIM parameters (*D*, *f*, D*, fD*) were evaluated. The selectivity of these changes was assessed using the mean of relative intermuscular changes.

### Statistical analysis

SPSS Statistics (version 29, IBM, Armonk, NY, USA) was used for all statistical analyses. The validity of assuming a normal distribution was examined with the Shapiro-Wilk test and the homogeneity of variances was evaluated with Levene’s test. Data are given as mean ± standard deviation for normally distributed data, or as median with interquartile range in parentheses. The Wilcoxon signed-rank test was performed to evaluate whether quantitative parameters differed before and after exercise-induced activation for each muscle; *p*-values were adjusted for multiple testing using the Bonferroni method; *p*-values ≤ 0.0125 were considered significant. Intraclass correlation coefficients for absolute agreement were calculated using a two-way mixed model to assess the reproducibility of IVIM parameters at rest between the three different MRI sessions; *p*-values below 0.05 were considered significant.

## Results

### Participants

The age of the ten study participants (five females and five males) was 32.0 ± 4.7 years (mean ± standard deviation). The body mass index was 23.0 ± 2.6 kg/m^2^. None of the volunteers were excluded during the study interval due to signs of fever, and no pathologies or incidental findings were identified on the anatomical MRI sequences.

### Quantitative image analysis

**Fig. 1:**
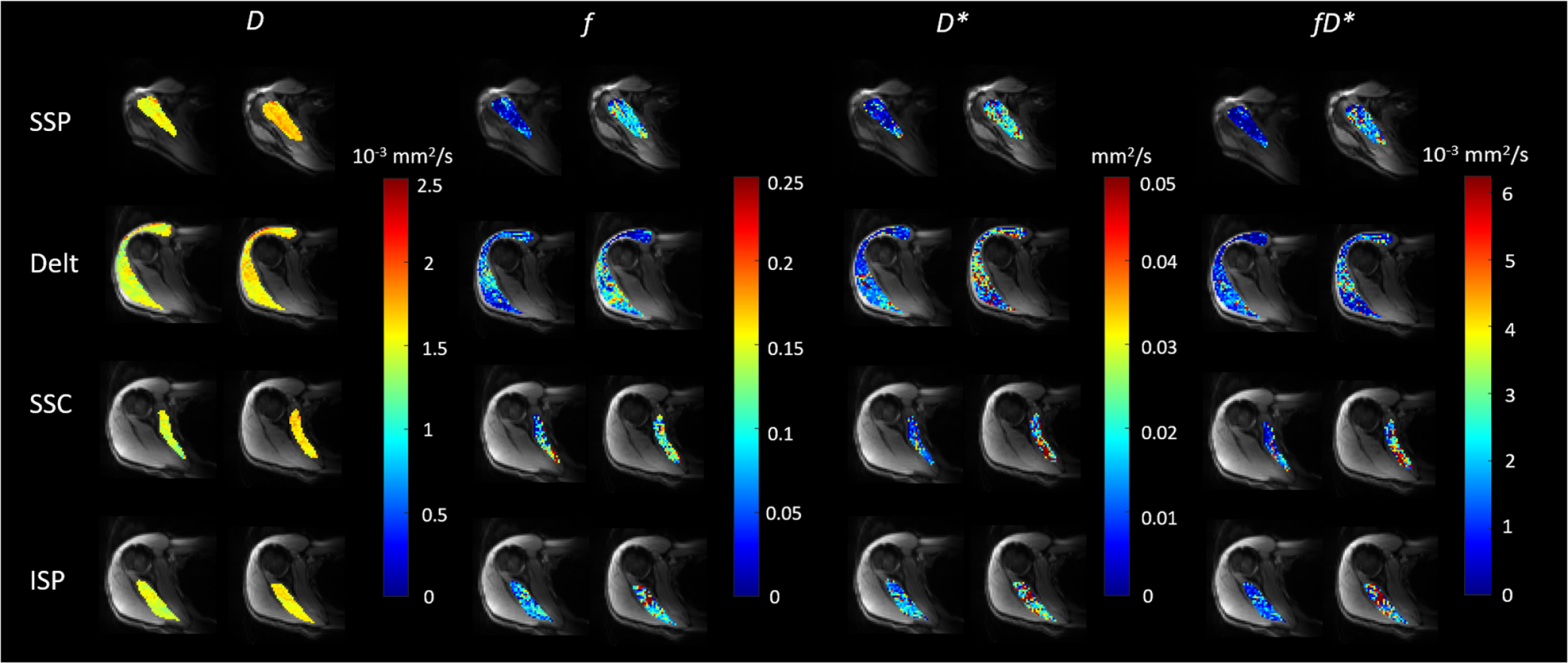
Representative images of a 32-year-old male participant: maps of the intravoxel incoherent motion parameters diffusion coefficient (*D*), perfusion fraction (*f*), perfusion-related pseudo-diffusion coefficient (D*), and blood-flow-related fD* before and after activation by the SSP test (Jobe test) are superimposed on transverse trace images. A significant increase of *D*, *f*, D*, and fD* was observed. Delt, Deltoid muscle; ISP, Infraspinatus muscle; SSC, Subscapularis muscle*;* SSP, Supraspinatus muscle

**Fig. 2:**
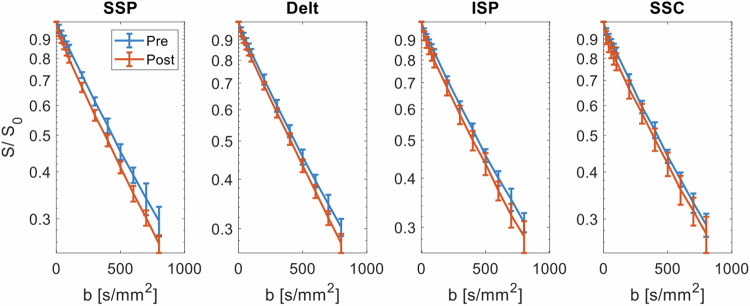
SSP test (Jobe test): signal decay averaged in the muscle region of interest and across all participants before the test (blue curve) and after the test (red curve). Error bars represent the standard deviation across subjects per *b*-value. Delt, Deltoid muscle; ISP, Infraspinatus muscle; SSC, Subscapularis muscle*;* SSP, Supraspinatus muscle

**Fig. 3:**
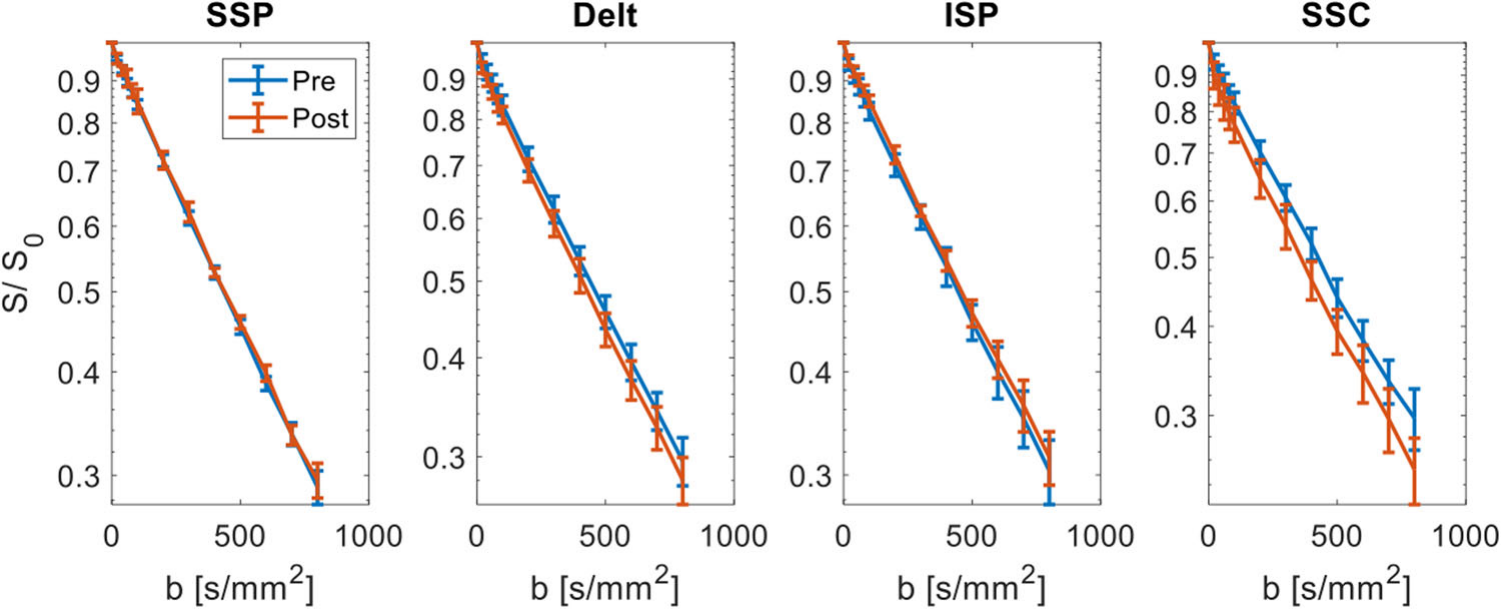
SSC test (lift-off test): signal decay averaged in the muscle region of interest and across all participants before the test (blue curve) and after the test (red curve). Error bars represent the standard deviation across subjects per *b*-value. Delt, Deltoid muscle; ISP, Infraspinatus muscle; SSC, Subscapularis muscle*;* SSP, Supraspinatus muscle

**Fig. 4:**
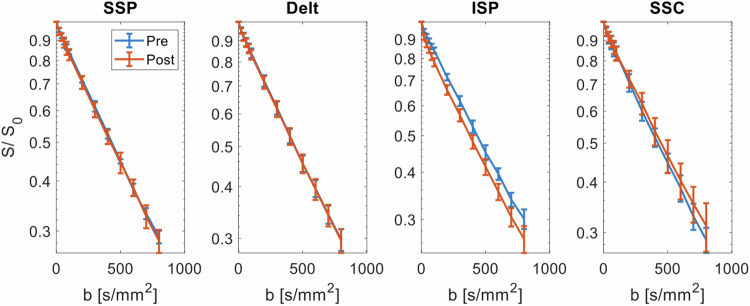
ISP test (external rotation test): signal decay averaged in the muscle region of interest and across all participants before the test (blue curve) and after the test (red curve). Error bars represent the standard deviation across subjects per *b*-value. Delt, Deltoid muscle; ISP, Infraspinatus muscle; SSC, Subscapularis muscle; SSP, Supraspinatus muscle

Baseline IVIM parameters of all three MRI sessions ranged from 1.42–1.54 [10^−3^ mm^2^ s^−1^] for *D*, 4.2–5.6% for *f*, and 15.1–19.0 [10^−3^ mm^2^ s^−1^] for D*. Intraclass correlation coefficients for baseline IVIM parameters of the three different MRI sessions were 0.876 (95% confidence interval (CI) 0.423–0.941) for *D*, 0.916 (95% CI 0.703–0.931) for *f*, and 0.859 (95% CI 0.661–0.903) for D*. IVIM parameter maps before and after the RC tests are presented for one participant in Fig. 1. The signal decay *versus* *b*-value for each test, averaged in the ROI and across all participants, is presented in Figs. 2–4. The SSP test induced a significant increase of all IVIM parameters in the SSP (*D*: 6.0%, *f*: 86.7%, D*: 52.5%, fD*: 186.3%; Table 2). Significant increases in IVIM parameters were also observed in the deltoid muscle (*D*: 6.2%, *f*: 70.8%, D*: 57.2%, fD*: 169.9%), in the SSC (*f*: 75.9%, D*: 42.1%, fD*: 149.5%), and in the ISP (*f*: 71.4%, D*: 51.7%, fD*: 160.7%).

**Table 2:** Diffusion coefficient *D*, perfusion fraction *f*, pseudo-diffusion coefficient D*, and blood-flow-related fD*, before and after performing the supraspinatus test

SSP (Jobe test)	Before activation	After activation	Relative difference (%)	*p*-value
SSP				
*D* [10^−3^ mm^2^ s^−1^]	1.51 (0.14)	1.60 (0.05)	6.0	0.010
*f* [%]	4.5 (3.0)	8.4 (2.2)	86.7	0.005
D* [10^−3^ mm^2^ s^−1^]	17.9 (9.3)	27.3 (7.2)	52.5	0.003
fD* [10^−3^ mm^2^ s^−1^]	0.80 (0.19)	2.29 (0.55)	186.3	< 0.001
Deltoid				
*D* [10^−3^ mm^2^ s^−1^]	1.45 (0.05)	1.54 (0.04)	6.2	0.010
*f* [%]	4.8 (2.6)	8.2 (1.6)	70.8	0.009
D* [10^−3^ mm^2^ s^−1^]	15.2 (5.5)	23.9 (12.0)	57.2	0.005
fD* [10^−3^ mm^2^ s^−1^]	0.73 (0.53)	1.97 (0.97)	169.9	< 0.001
SSC				
*D* [10^−3^ mm^2^ s^−1^]	1.50 (0.08)	1.50 (0.11)	0.0	1.000
*f* [%]	5.4 (3.5)	9.5 (4.6)	75.9	0.005
D* [10^−3^ mm^2^ s^−1^]	19.0 (9.2)	27.1 (13.6)	42.1	0.008
fD* [10^−3^ mm^2^ s^−1^]	1.03 (0.19)	2.57 (0.43)	149.5	< 0.001
ISP				
*D* [10^−3^ mm^2^ s^−1^]	1.42 (0.09)	1.47 (0.08)	3.5	0.013
*f* [%]	5.6 (2.8)	9.6 (4.0)	71.4	0.007
D* [10^−3^ mm^2^ s^−1^]	15.1 (5.0)	22.9 (10.2)	51.7	0.007
fD* [10^−3^ mm^2^ s^−1^]	0.84 (0.32)	2.19 (0.66)	160.7	< 0.001

Data are given as median with interquartile range in parentheses. The *p*-values were obtained by applying the Wilcoxon signed-rank test with Bonferroni correction for multiple testing; *p*-values ≤ 0.0125 were considered significant

*ISP* Infraspinatus muscle, *SSC* Subscapularis muscle, *SSP* Supraspinatus muscle

Following the SSC test, all IVIM parameters in the SSC were significantly increased (*D*: 6.8%, *f*: 117.3%, D*: 47.5%, fD*: 221.0%; Table 3), whereas there were no significant changes in the other muscles (deltoid, SSP, and ISP). For the ISP test, a similar activation pattern was observed: whereas all IVIM parameters in the ISP were significantly larger after activation (*D*: 4.1%, *f*: 108.5%, D*: 58.4%, fD*: 229.2%; Table 4), the changes in the other muscles (deltoid, SSP, and SSC) were non-significant.

**Table 3:** Diffusion coefficient *D*, perfusion fraction *f*, pseudo-diffusion coefficient D*, and blood-flow-related fD* before and after performing the subscapularis test

SSC (Lift-off test)	Before activation	After activation	Relative difference (%)	*p*-value
SSP				
*D* [10^−3^ mm^2^ s^−1^]	1.53 (0.06)	1.50 (0.05)	2.0	1.000
*f* [%]	4.3 (2.2)	4.6 (2.3)	7.0	1.000
D* [10^−3^ mm^2^ s^−1^]	17.8 (7.5)	14.8 (8.4)	16.9	0.169
fD* [10^−3^ mm^2^ s^−1^]	0.77 (0.22)	0.68 (0.24)	11.7	0.152
Deltoid				
*D* [10^−3^ mm^2^ s^−1^]	1.47 (0.06)	1.51 (0.06)	2.7	0.059
*f* [%]	4.2 (2.3)	4.9 (2.1)	16.7	0.028
D* [10^−3^ mm^2^ s^−1^]	17.3 (9.1)	20.0 (10.5)	15.6	0.028
fD* [10^−3^ mm^2^ s^−1^]	0.72 (0.25)	0.98 (0.35)	36.1	0.016
SSC				
*D* [10^−3^ mm^2^ s^−1^]	1.48 (0.09)	1.58 (0.10)	6.8	0.009
*f* [%]	5.2 (3.5)	11.3 (5.0)	117.3	0.007
D* [10^−3^ mm^2^ s^−1^]	18.3 (11.7)	27.0 (13.3)	47.5	0.001
fD* [10^−3^ mm^2^ s^−1^]	0.95 (0.27)	3.05 (0.40)	221.0	< 0.001
ISP				
*D* [10^−3^ mm^2^ s^−1^]	1.43 (0.11)	1.41 (0.10)	1.4	1.000
*f* [%]	5.2 (3.5)	6.0 (5.1)	15.3	0.055
D* [10^−3^ mm^2^ s^−1^]	17.9 (7.8)	19.3 (10.6)	7.8	0.636
fD* [10^−3^ mm^2^ s^−1^]	0.93 (0.21)	1.16 (0.31)	24.7	0.008

Data are given as median with interquartile range in parentheses. The *p*-values were obtained by applying the Wilcoxon signed-rank test with Bonferroni correction for multiple testing; *p*-values ≤ 0.0125 were considered significant

*ISP* Infraspinatus muscle, *SSC* Subscapularis muscle, *SSP* Supraspinatus muscle

**Table 4:** Diffusion coefficient *D*, perfusion fraction *f*, pseudo-diffusion coefficient D*, and blood-flow-related fD* before and after performing the infraspinatus test

ISP (External rotation test)	Before activation	After activation	Relative difference (%)	*p*-value
SSP				
*D* [10^−3^ mm^2^ s^−1^]	1.54 (0.05)	1.53 (0.07)	0.6	1.000
*f* [%]	4.2 (2.3)	4.1 (3.6)	2.4	1.000
D* [10^−3^ mm^2^ s^−1^]	15.4 (7.1)	17.3 (8.9)	12.3	0.203
fD* [10^−3^ mm^2^ s^−1^]	0.64 (0.42)	0.71 (0.44)	10.9	0.826
Deltoid				
*D* [10^−3^ mm^2^ s^−1^]	1.49 (0.05)	1.48 (0.06)	0.7	1.000
*f* [%]	3.5 (2.5)	3.9 (2.6)	10.3	0.225
D* [10^−3^ mm^2^ s^−1^]	17.9 (8.1)	18.3 (10.1)	2.2	1.000
fD* [10^−3^ mm^2^ s^−1^]	0.63 (0.21)	0.71 (0.37)	12.7	0.225
SSC				
*D* [10^−3^ mm^2^ s^−1^]	1.51 (0.08)	1.44 (0.15)	4.6	0.738
*f* [%]	5.0 (4.7)	4.6 (3.4)	8.0	0.924
D* [10^−3^ mm^2^ s^−1^]	15.5 (8.3)	16.1 (6.7)	3.9	1.000
fD* [10^−3^ mm^2^ s^−1^]	0.78 (0.29)	0.74 (0.49)	5.1	0.828
ISP				
*D* [10^−3^ mm^2^ s^−1^]	1.48 (0.07)	1.54 (0.06)	4.1	0.007
*f* [%]	4.7 (2.2)	9.8 (1.5)	108.5	0.005
D* [10^−3^ mm^2^ s^−1^]	19.0 (8.7)	30.1 (12.8)	58.4	0.007
fD* [10^−3^ mm^2^ s^−1^]	0.89 (0.22)	2.93 (0.46)	229.2	0.001

Data are given as median with interquartile range in parentheses. The *p*-values were obtained by applying the Wilcoxon signed-rank test with Bonferroni correction for multiple testing; *p*-values ≤ 0.0125 were considered significant

*ISP* Infraspinatus muscle, *SSC* Subscapularis muscle, *SSP* Supraspinatus muscle

## Discussion

This study aimed to measure regional changes of IVIM parameters in the RC muscles induced by three different physical RC muscle tests to explore if the muscles of the surrounding shoulder girdle muscles were coactivated after the respective clinical test.

The first main finding was that the SSP test did not selectively activate the SSP muscle, as coactivation of the deltoid, SSC, and ISP muscles was observed.

It is well known that physical activity causes a global redistribution of blood-flow in favor of the active muscle group [25]. IVIM has been successfully used to investigate changes in muscle perfusion following activation, demonstrating the capability to identify the specific muscle involved in a particular task [20]. In the present study, IVIM parameters observed at rest and after activation were largely consistent with the findings of Nguyen et al [21, 22], which may highlight the reproducibility of IVIM for the assessment of muscle activation in the shoulder girdle. Differences between the aforementioned and the present study could be explained by variations in imaging protocol parameters, as IVIM parameters are heavily dependent on tissue relaxation properties [26].

Our results are also in agreement with EMG studies that report coactivation of these muscles in the Jobe position when testing the SSP [12–14] and are supported by the knowledge that during scapular plane abduction, the deltoid has been identified as the principal movement driver, while the SSC and ISP are activated to stabilize the humeral head against the glenoid, providing a fulcrum for the deltoid in the early phases of abduction [13, 27].

Numerous previous studies have found that SSP testing for pathologies has an unsatisfactory diagnostic accuracy: in a review by Longo et al, the Jobe test was found to have a sensitivity of less than 80% in four of six studies and a specificity of less than 80% in five of six studies [28]. On the other hand, the anatomical validity of the lift-off test for SSC testing has been evaluated and confirmed [15, 29], while the improved diagnostic performance might be attributed to the fact that the lift-off test isolates the SSC from the internal rotating and adducting forces of the pectoralis major [30].

In contrast to our study, a coactivation of the posterior deltoid was observed during the lift-off test in a study by Nguyen et al [22]. The authors hypothesized that this may be caused by the requirement for some degree of shoulder extension in the lift-off position. These differences may be caused by the fact that this study evaluated the ROI average of IVIM parameters for the entire deltoid muscle rather than separately analyzing distinct muscle regions.

In the present study, only the ISP was activated during the seated external rotation test, while no activation of the deltoid or other RC muscles was recorded. Although there are conflicting results in the current literature regarding RC coactivation during external rotation for ISP testing [8, 31], the seated position is preferred over the supine position to minimize middle and posterior deltoid muscle activity [32], which is in line with our results and may be due to the lower capsular strain and muscle tension in the seated position compared to other testing positions [31, 32]. Moreover, a towel roll was used in our study, which is supposed to ensure a proper technique for the participant to minimize shoulder abduction while externally rotating the shoulder [31].

This study has several limitations. First, the sample size of this investigation was small, which limits the generalizability of our findings. Second, a delay between test execution and MRI acquisition was inevitable, even though it was minimized by performing the tests in the MRI examination room. Third, only healthy volunteers were evaluated in this study.

In conclusion, this study used IVIM to demonstrate selective muscle activation in the RC after performing different clinical shoulder examinations. An isolated activity increase of the SSC and ISP was observed after a lift-off and external rotation test, while for the SSP, scattered activation patterns of the surrounding RC muscles after the Jobe test were found. Our results suggest limited anatomical specificity for the Jobe test to target the SSP, while tests for the SSC and ISP showed improved isolation of the target muscle. Our results aligned with results from prior studies that studied activation-related changes of the RC after physical testing, which corroborates the reproducibility of the IVIM technique for functional imaging of the RC muscles. As a future perspective, IVIM may be useful for a comprehensive assessment of how patient positioning affects RC muscle activation, with the advantage of being noninvasive and providing spatial information on the entire RC rather than a small number of fibers as in EMG. This may facilitate the design of more effective clinical RC tests or the identification of activation patterns that may influence the outcome after RC repair.

## Data Availability

The datasets used and/or analyzed during the current study are available from the corresponding author upon reasonable request.

## Abbreviations

CI: Confidence interval
DWI: Diffusion-weighted Imaging
EMG: Electromyography
ISP: Infraspinatus muscle
IVIM: Intravoxel incoherent motion
MRI: Magnetic resonance imaging
RC: Rotator cuff
ROI: Region of interest
SSC: Subscapularis muscle
SSP: Supraspinatus muscle

## References

[1] Colvin AC, Egorova N, Harrison AK, Moskowitz A, Flatow EL (2012) National trends in rotator cuff repair. J Bone Joint Surg Am 94:227–233. 10.2106/jbjs.J.00739 22298054 10.2106/jbjs.J.00739 PMC3262185

[2] Yamamoto A, Takagishi K, Osawa T et al (2010) Prevalence and risk factors of a rotator cuff tear in the general population. J Shoulder Elbow Surg 19:116–120. 10.1016/j.jse.2009.04.006 19540777 10.1016/j.jse.2009.04.006

[3] Murrell GAC, Walton JR (2001) Diagnosis of rotator cuff tears. Lancet 357:769–770. 10.1016/S0140-6736(00)04161-1 11253973 10.1016/S0140-6736(00)04161-1

[4] Jobe FW, Moynes DR (1982) Delineation of diagnostic criteria and a rehabilitation program for rotator cuff injuries. Am J Sports Med 10:336–339. 10.1177/036354658201000602 7180952 10.1177/036354658201000602

[5] Fieseler G, Laudner K, Cornelius J, Schulze S, Delank KS, Schwesig R (2023) Longitudinal analysis of the ASES and constant-murley scores, and the internal rotation/shift and jobe tests following arthroscopic repair of supraspinatus lesions. J Pers Med 13:1304. 10.3390/jpm13091304 37763072 10.3390/jpm13091304 PMC10533080

[6] Itoi E, Minagawa H, Yamamoto N, Seki N, Abe H (2006) Are pain location and physical examinations useful in locating a tear site of the rotator cuff? Am J Sports Med 34:256–264. 10.1177/0363546505280430 16219939 10.1177/0363546505280430

[7] Gerber C, Krushell RJ (1991) Isolated rupture of the tendon of the subscapularis muscle. Clinical features in 16 cases. J Bone Joint Surg Br 73:389–394. 10.1302/0301-620x.73b3.1670434 1670434 10.1302/0301-620x.73b3.1670434

[8] Clisby EF, Bitter NL, Sandow MJ, Jones MA, Magarey ME, Jaberzadeh S (2008) Relative contributions of the infraspinatus and deltoid during external rotation in patients with symptomatic subacromial impingement. J Shoulder Elbow Surg 17:87–92. 10.1016/j.jse.2007.05.019 10.1016/j.jse.2007.05.019 18162415

[9] Chalmers PN, Cvetanovich GL, Kupfer N et al (2016) The champagne toast position isolates the supraspinatus better than the Jobe test: an electromyographic study of shoulder physical examination tests. J Shoulder Elbow Surg 25:322–329. 10.1016/j.jse.2015.07.031 26443105 10.1016/j.jse.2015.07.031

[10] Hughes PC, Taylor NF, Green RA (2008) Most clinical tests cannot accurately diagnose rotator cuff pathology: a systematic review. Aust J Physiother 54:159–170. 10.1016/s0004-9514(08)70022-9 18721119 10.1016/s0004-9514(08)70022-9

[11] Katepun S, Boonsun P, Boonsaeng WS, Apivatgaroon A (2023) Reliability of the single-arm and double-arm jobe test for the diagnosis of full-thickness supraspinatus tendon tear. Orthop J Sports Med 11:23259671231187631. 10.1177/23259671231187631 37547080 10.1177/23259671231187631 PMC10402285

[12] Reddy AS, Mohr KJ, Pink MM, Jobe FW (2000) Electromyographic analysis of the deltoid and rotator cuff muscles in persons with subacromial impingement. J Shoulder Elbow Surg 9:519–523. 10.1067/mse.2000.109410 11155306 10.1067/mse.2000.109410

[13] Alpert SW, Pink MM, Jobe FW, McMahon PJ, Mathiyakom W (2000) Electromyographic analysis of deltoid and rotator cuff function under varying loads and speeds. J Shoulder Elbow Surg 9:47–58. 10.1016/s1058-2746(00)90009-0 10717862 10.1016/s1058-2746(00)90009-0

[14] Malanga GA, Jenp YN, Growney ES, An KN (1996) EMG analysis of shoulder positioning in testing and strengthening the supraspinatus. Med Sci Sports Exerc 28:661–664. 10.1097/00005768-199606000-00003 8784752 10.1097/00005768-199606000-00003

[15] Greis PE, Kuhn JE, Schultheis J, Hintermeister R, Hawkins R (1996) Validation of the lift-off test and analysis of subscapularis activity during maximal internal rotation. Am J Sports Med 24:589–593. 10.1177/036354659602400505 8883677 10.1177/036354659602400505

[16] Morris AD, Kemp GJ, Lees A, Frostick SP (1998) A study of the reproducibility of three different normalisation methods in intramuscular dual fine wire electromyography of the shoulder. J Electromyogr Kinesiol 8:317–322. 10.1016/s1050-6411(98)00002-9 9785252 10.1016/s1050-6411(98)00002-9

[17] Kinugasa R, Kawakami Y, Fukunaga T (2006) Quantitative assessment of skeletal muscle activation using muscle functional MRI. Magn Reson Imaging 24:639–644. 10.1016/j.mri.2006.01.002 16735187 10.1016/j.mri.2006.01.002

[18] Bihan DL, Turner R (1992) The capillary network: a link between IVIM and classical perfusion. Magn Reson Med 27:171–178. 10.1002/mrm.1910270116 1435202 10.1002/mrm.1910270116

[19] Le Bihan D (2019) What can we see with IVIM MRI? Neuroimage 187:56–67. 10.1016/j.neuroimage.2017.12.062 29277647 10.1016/j.neuroimage.2017.12.062

[20] Ohno N, Miyati T, Fujihara S, Gabata T, Kobayashi S (2020) Biexponential analysis of intravoxel incoherent motion in calf muscle before and after exercise: comparisons with arterial spin labeling perfusion and T2. Magn Reson Imaging 72:42–48. 10.1016/j.mri.2020.06.003 32561379 10.1016/j.mri.2020.06.003

[21] Nguyen A, Ledoux J-B, Omoumi P, Becce F, Forget J, Federau C (2017) Selective microvascular muscle perfusion imaging in the shoulder with intravoxel incoherent motion (IVIM). Magn Reson Imaging 35:91–97. 10.1016/j.mri.2016.08.005 27576020 10.1016/j.mri.2016.08.005

[22] Nguyen A, Ledoux JB, Omoumi P, Becce F, Forget J, Federau C (2016) Application of intravoxel incoherent motion perfusion imaging to shoulder muscles after a lift-off test of varying duration. NMR Biomed 29:66–73. 10.1002/nbm.3449 26684052 10.1002/nbm.3449

[23] Hughes PC, Green RA, Taylor NF (2014) Isolation of infraspinatus in clinical test positions. J Sci Med Sport 17:256–260. 10.1016/j.jsams.2013.05.011 23809837 10.1016/j.jsams.2013.05.011

[24] Le Bihan D, Breton E, Lallemand D, Aubin ML, Vignaud J, Laval-Jeantet M (1988) Separation of diffusion and perfusion in intravoxel incoherent motion MR imaging. Radiology 168:497–505. 10.1148/radiology.168.2.3393671 3393671 10.1148/radiology.168.2.3393671

[25] Smith JAB, Murach KA, Dyar KA, Zierath JR (2023) Exercise metabolism and adaptation in skeletal muscle. Nat Rev Mol Cell Biol 24:607–632. 10.1038/s41580-023-00606-x 37225892 10.1038/s41580-023-00606-x PMC10527431

[26] Adelnia F, Shardell M, Bergeron CM et al (2019) Diffusion-weighted MRI with intravoxel incoherent motion modeling for assessment of muscle perfusion in the thigh during post-exercise hyperemia in younger and older adults. NMR Biomed 32:e4072. 10.1002/nbm.4072 30861224 10.1002/nbm.4072 PMC6530599

[27] Inman VT, Saunders JdM, Abbott LC (1944) Observations on the function of the shoulder joint. J Bone Joint Surg Am 26:1–30. 10.1097/00003086-199609000-00002 10.1097/00003086-199609000-00002

[28] Longo UG, Berton A, Ahrens PM, Maffulli N, Denaro V (2011) Clinical tests for the diagnosis of rotator cuff disease. Sports Med Arthrosc Rev 19:266–278. 10.1097/JSA.0b013e3182250c8b 21822110 10.1097/JSA.0b013e3182250c8b

[29] Tokish JM, Decker MJ, Ellis HB, Torry MR, Hawkins RJ (2003) The belly-press test for the physical examination of the subscapularis muscle: electromyographic validation and comparison to the lift-off test. J Shoulder Elbow Surg 12:427–430. 10.1016/s1058-2746(03)00047-8 14564261 10.1016/s1058-2746(03)00047-8

[30] Bartsch M, Greiner S, Haas NP, Scheibel M (2010) Diagnostic values of clinical tests for subscapularis lesions. Knee Surg Sports Traumatol Arthrosc 18:1712–1717. 10.1007/s00167-010-1109-1 20376624 10.1007/s00167-010-1109-1

[31] Reinold MM, Wilk KE, Fleisig GS et al (2004) Electromyographic analysis of the rotator cuff and deltoid musculature during common shoulder external rotation exercises. J Orthop Sports Phys Ther 34:385–394. 10.2519/jospt.2004.34.7.385 15296366 10.2519/jospt.2004.34.7.385

[32] Kim J-W, Yoon J-Y, Kang M-H, Oh J-S (2012) Selective activation of the infraspinatus during various shoulder external rotation exercises. J Phys Ther Sci 24:581–584. 10.1589/jpts.24.581 10.1589/jpts.24.581

